# Settling for second best: when should doctors agree to parental demands for suboptimal medical treatment?

**DOI:** 10.1136/medethics-2016-103461

**Published:** 2017-09-25

**Authors:** Tara Nair, Julian Savulescu, Jim Everett, Ryan Tonkens, Dominic Wilkinson

**Affiliations:** 1 Faculty of Medicine, Nursing and Health Sciences, Monash University, Melbourne, Victoria, Australia; 2 Oxford Uehiro Centre for Practical Ethics, Faculty of Philosophy, University of Oxford, Oxford, UK; 3 Department of Experimental Psychology, University of Oxford, Oxford, UK; 4 Centre for Human Bioethics, Monash University, Melbourne, Victoria, Australia; 5 Department of Paediatrics, John Radcliffe Hospital, Oxford, UK

**Keywords:** newborns and minors, paediatrics, decision-making, demographic surveys/attitudes, right to refuse treatment

## Abstract

**Background:**

Doctors sometimes encounter parents who object to prescribed treatment for their children, and request suboptimal substitutes be administered instead (suboptimal being defined as less effective and/or more expensive). Previous studies have focused on parental refusal of treatment and when this should be permitted, but the ethics of requests for suboptimal treatment has not been explored.

**Methods:**

The paper consists of two parts: an empirical analysis and an ethical analysis. We performed an online survey with a sample of the general public to assess respondents’ thresholds for acceptable harm and expense resulting from parental choice, and the role that religion played in their judgement. We also identified and applied existing ethical frameworks to the case described in the survey to compare theoretical and empirical results.

**Results:**

Two hundred and forty-two Mechanical Turk workers took our survey and there were 178 valid responses (73.6%). Respondents’ agreement to provide treatment decreased as the risk or cost of the requested substitute increased (p<0.001). More than 50% of participants were prepared to provide treatment that would involve a small absolute increased risk of death for the child (<5%) and a cost increase of US$<500, respectively. Religiously motivated requests were significantly more likely to be allowed (p<0.001). Existing ethical frameworks largely yielded ambiguous results for the case. There were clear inconsistencies between the theoretical and empirical results.

**Conclusion:**

Drawing on both survey results and ethical analysis, we propose a potential model and thresholds for deciding about the permissibility of suboptimal treatment requests.

## Introduction

Patients’ opinions, beliefs and values hold an important place in clinical decision making in modern medicine.^1^ A competent patient may refuse treatment for themselves on religious or non-religious grounds and a doctor must respect this decision, even if they consider it to be irrational.^2^ However, this is more complicated when the individual is making decisions on behalf of another (eg, a parent making treatment decisions for their child). Parents’ right to make medical treatment decisions on behalf of their child is not unlimited.^3^

There has been research into the ethics of parental refusal of treatment, for example, Jehovah’s Witness parents who refuse a blood transfusion for their children. In cases where the life of the child is at risk, it is widely accepted that doctors should over-ride parents’ wishes.^3^ But the question of what doctors should do when parents are not refusing treatment, rather requesting a substitute ‘second best’ treatment, has attracted less attention. Box 1 describes a paradigm example of such a case.

Box 1 **An example request for substitute suboptimal treatment. (A substitute treatment is defined as suboptimal if it is less effective and/or more costly than the standard treatment)**

A very premature baby has just been born. The neonate is seriously ill and in intensive care with respiratory distress syndrome (RDS), a common complication of prematurity^40^ ^41^ caused by a deficiency of surfactant. Surfactant replacement therapy (SRT) is a highly effective treatment for RDS, which reduces the risk of air leak, pneumothorax, pulmonary interstitial emphysema, bronchopulmonary dysplasia and neonatal mortality.^13^ However, the parents of this child have an objection to the prescribed medication because they are Muslim and the medication contains pork-derived ingredients which they consider haram (forbidden by Allah).^42^ (This medication may also receive objections from Jewish parents,^43^ as well as parents who are vegetarian, vegan or have an interest in animal rights.^44^) The parents have requested a substitute preparation of surfactant. There is a bovine-derived surfactant preparation which is both less effective^39 45^ and more expensive,^38^ and a synthetic preparation which may potentially be equally effective^46 47^ but is significantly more expensive than both the porcine and bovine-derived preparations.^48^

Previous empirical research indicates that professionals are unsure how to respond to parental choice around treatments in cases like the one described in box 1. Two surveys of UK and US doctors indicated that professionals did not usually discuss the constituents of surfactant replacement with parents of premature newborns.^4 5^ However, in another survey, when asked from the perspective of a parent, a majority of medical and non-medical staff (74%) wanted the different types of surfactant replacement to be discussed before administration and 79% said that the hospital should stock at least one substitute preparation.^6^

Numerous pressing ethical questions emerge from cases like this. First, is it permissible for parents to choose substitute medical treatments that are *less effective* than those recommended by doctors? If so, how much less effective? Second, is it permissible in a public healthcare system for parents to choose more expensive medical treatments than those recommended by doctors? If so, what additional cost is acceptable? And third, does the reason behind the parents’ choice make a difference?

Normative models have been developed that can be used to assess whether or not it is justified for a physician to over-ride parents’ medical decision making for their child.^7–9^ However, these models have not been specifically applied to requests for suboptimal treatment.

The aim of this study was to explore the boundaries of parental choice and identify thresholds of acceptable levels of harm and cost using both empirical and theoretical methods. In the first part of the paper we perform an empirical test of the general public’s intuitions about permissible levels of harm and expense caused by parental choice. In the second part of the paper we discuss existing ethical frameworks and compare and contrast the data gathered from our survey with the suggestions offered by ethical theory to aim at reflective equilibrium.

## Part 1: Empirical analysis

### Methods

#### Participants and procedure

American participants (n=242) were recruited online through Amazon Mechanical Turk (MTurk) and paid $1 for their time. MTurk is a website that facilitates payment for completing Human Intelligence Tasks (HITs) posted by researchers, and is widely used in social science research because it provides participants who vary in age, socioeconomic status and ethnic background^10^ and are broadly representative of the US population. A power analysis revealed that a minimum of 128 participants was required to detect a moderate effect size (d=0.5) at the 5% level with 80% chance. Participants were excluded from data analysis if they failed to complete the survey (n=33) or failed a simple attention check^11^ (n=31). Thus, our final sample used in analysis consisted of 178 participants.

Participants completed the study through the online survey tool Qualtrics. The survey was piloted for face validity with a cohort of students and colleagues, and the order of the questions within all sections was randomised to reduce order effect.^12^ Ethics approval for this study was provided by the Social Sciences and Humanities Inter-Divisional Research Ethics Committee of the University of Oxford.

#### Design

We hypothesised that when faced with a request for a substitute medical treatment, members of the public would be increasingly inclined to refuse parents’ requests as the comparative efficacy decreases, and as the expense increases. We predicted that survey respondents would be more likely to allow requests based on religious rather than non-religious reasons.

#### Sections 1 and 2

**Figure 1:**
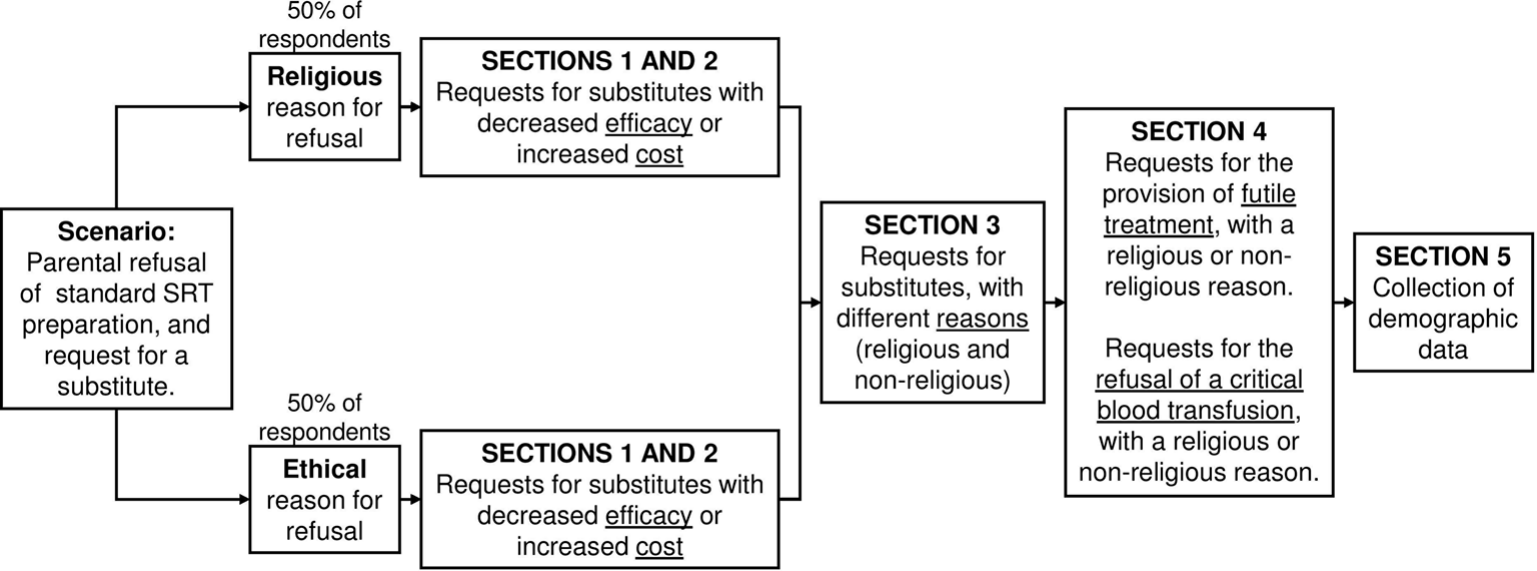
Flow chart of survey structure (see full survey; Appendix A). SRT, surfactant replacement therapy.

The first two sections of the study had a mixed-subjects design: a between-subjects factor of whether the reasons for requesting the substitute treatment were ‘religious’ or ‘ethical’; and a within-subjects factor of whether this treatment was less efficacious or more expensive (figure 1).

10.1136/medethics-2016-103461.supp1 Supplementary file

Respondents were asked to imagine themselves as health professionals facing a request for substitute treatment (using the example of surfactant replacement). Participants were informed that without treatment the infant was stated to have a 30% absolute risk of mortality, and with the standard form of treatment this risk was reduced to 15% (these figures were based on data^13^ on the effectiveness of surfactant replacement therapy, but slightly exaggerated and simplified for the sake of clarity). Respondents were randomised to receive one of two versions of these sections of the survey; 50% were told the parents’ reason for objecting was ‘religious’ and the other 50% were told the parents’ reason for objecting was ‘ethical.’

**Figure 2:**
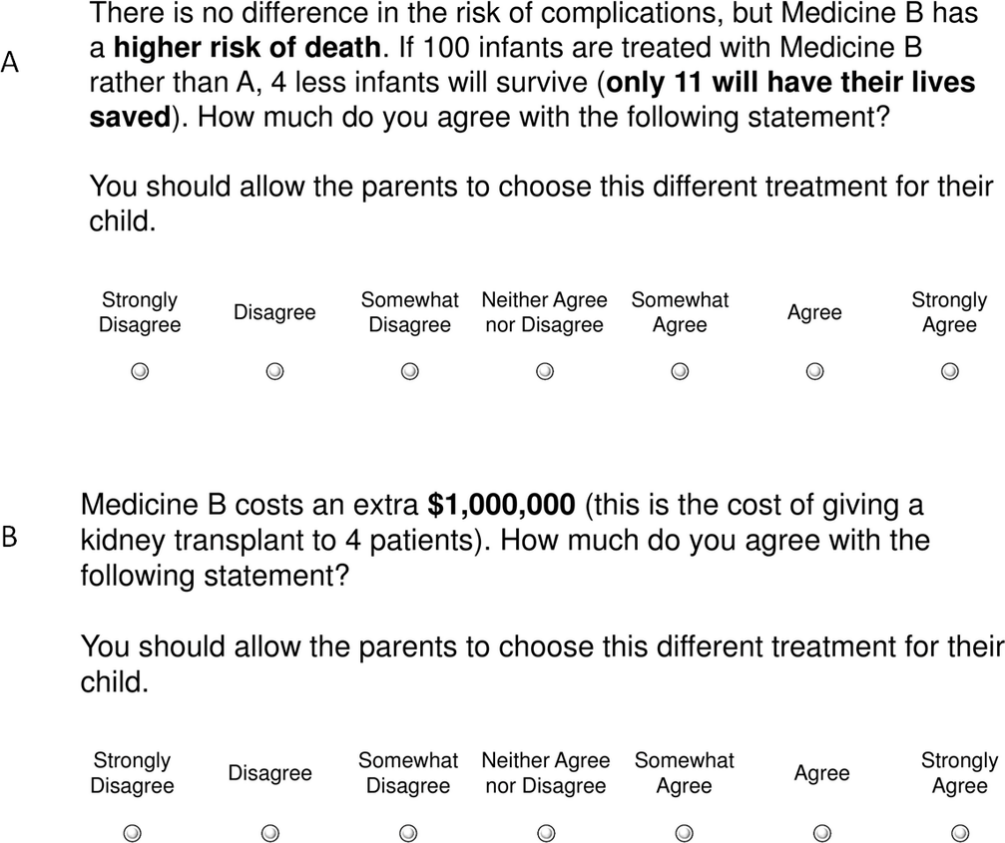
(A) Sample question from the survey, investigating willingness to provide less effective treatment. (B) Sample question from the survey, investigating willingness to provide more expensive treatment.

To look at the influence of efficacy and expense on support for substitute treatments, we asked participants to respond to a series of variations of the case example. First, participants were told that the hypothetical parents had either refused treatment or had requested treatments with varying degrees of reduced efficacy. The substitute treatments had a higher risk of pneumothorax needing drainage (higher risk of a painful procedure), intraventricular haemorrhage (higher risk of disability) or of death (4%, 9% or 14% absolute increase in risk: see figure 2A). Next, participants were told that the substitute treatments requested were equally effective, but associated with varying degrees of additional cost (see figure 2B). To provide context for the cost of treatment, indicative examples were provided of other medical treatments with similar expense.^14^ Respondents were asked to indicate their level of agreement with allowing these parental requests on a 7-point Likert scale (1=strongly disagree, 7=strongly agree).

#### Section 3

This part of the study assessed all participants’ agreement to different *reasons* behind parental requests. There were five versions in a within-subjects design presented to all participants: three religious reasons for objection (Muslim, Christian and Jewish parents) and two non-religious reasons (vegan parents and parents with an irrational fear their child will develop an allergy to the medication). For each, respondents were asked whether they would agree to provide either a less effective substitute (with a higher, but unspecified chance of death) or a more expensive substitute (double the cost of the standard treatment).

#### Section 4

In order to allow comparison between requests for suboptimal treatment and other examples of parental discretion around treatment, the fourth part of the survey assessed respondents’ willingness to accede to two other widely discussed controversial parental requests. They were asked if they would agree to prolong treatment deemed futile by medical professionals, or allow parents to decline a blood transfusion in a severely anaemic child. For each case, the respondents were provided with two versions, one where the parents’ request was based on a religious reason and the other on a non-religious reason.

#### Section 5

Finally, to look at how beliefs about the acceptability of suboptimal treatments might differ across different groups, we collected basic demographic information. Given that some of the reasons the hypothetical parents gave for refusing treatment were religious ones, we measured how religious participants considered themselves using the Central Religiosity Scale (CRS).^15^ The CRS (α=0.93) consists of five items tapping different dimensions of religious belief (eg, private practice, public practice and religious experience). Similarly, because some of the reasons the hypothetical parents gave were based on ethical views relating to the use of animals, we measured participants’ concern with animal welfare using an 8-itemed Speciesism Scale^i^ (α=0.87).

Statistical analysis was conducted using SPSS Statistics V.22 for Windows (IBM). Paired samples t-tests were used to compare the participants’ responses between questions. Independent samples t-tests were used to investigate the differences between the participants given religious-based requests and those given non-religious versions. A p value of <0.05 was considered significant.

## Results

### Participants

The majority of the respondents were between 25 and 34 years old, and there were more female participants (Appendix B). Forty-one per cent had a tertiary education and three quarters of respondents were employed. All resided in the USA, but nearly 40% originated elsewhere. Just over half of the cohort had a religious affiliation (the most common religion was Christianity), while over a third considered themselves atheists. The CRS showed approximately half of the cohort to be ‘Religious’ or ‘Highly Religious’ (Appendix C). Eighty per cent had no dietary preference.

### Sections 1 and 2

**Figure 3:**
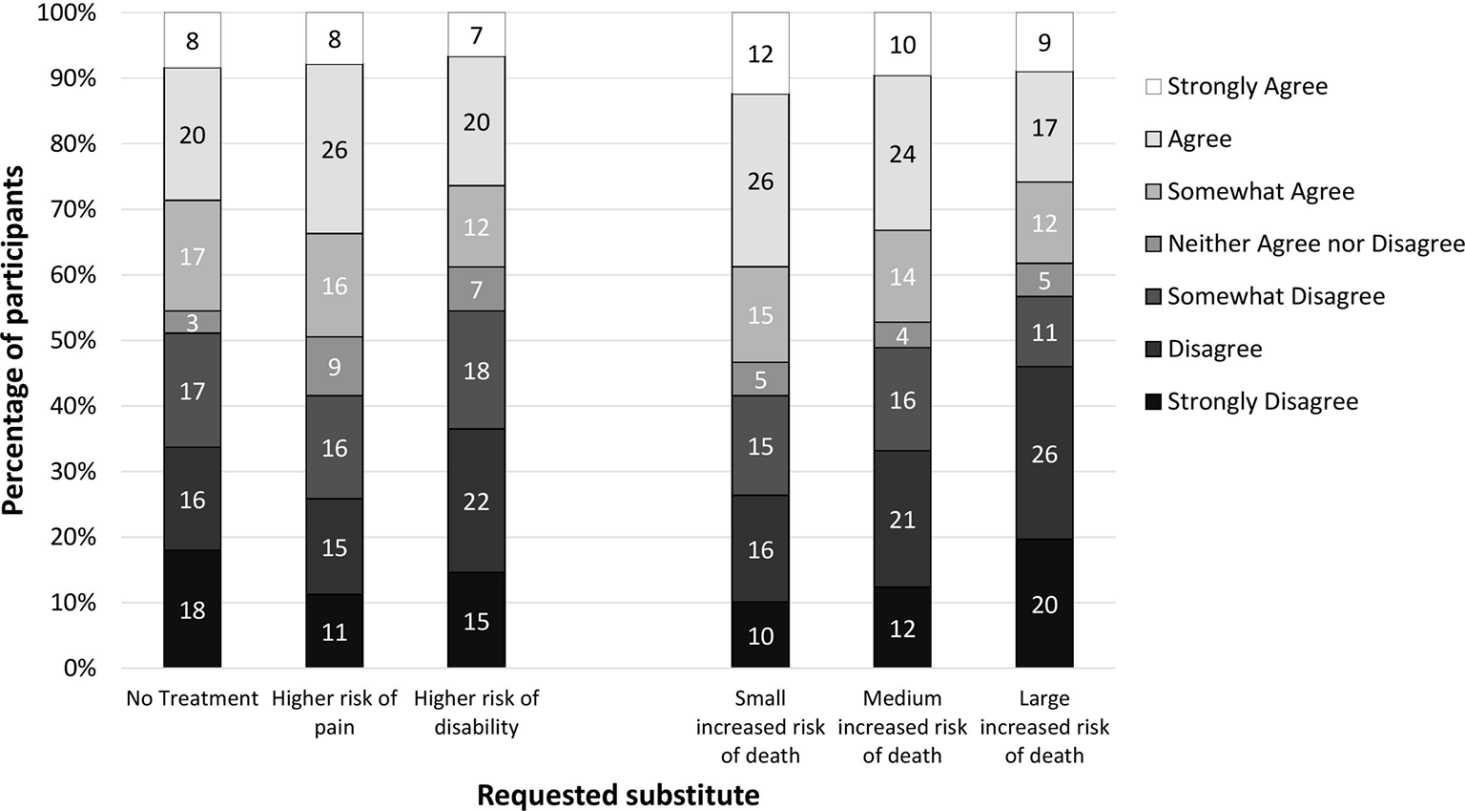
Level of agreement to provide no treatment or substitute treatments of reduced efficacy.

When given scenarios of parents requesting less effective treatment, the majority of respondents agreed to provide substitute treatment with a small increased mortality risk (19% absolute mortality risk). Participants were less inclined to provide treatment that would increase a child’s risk of disability (mean agreement score (M)=3.66, SD=1.95) compared with a treatment that would increase the risk of pain (M=4.12, SD=1.91) (t(177)=4.78, p<0.001), indicating that participants were more concerned about whether the treatment would cause disability than whether it would cause pain (figure 3). Participants were significantly less likely to provide treatment as the increased absolute risk of death from the substitute treatment went from small (+4%) to medium (+9%) to large (+14%) (*F*(2,354)=37.94, p<0.001).

Twenty-eight per cent of respondents strongly agreed or agreed to allow parental refusal of treatment (30% absolute mortality, M=3.8, SD=2.04). Of note, this was higher than the level of agreement to provide a substitute treatment associated with a large increased risk of death (lower risk than that of no treatment; 29% absolute mortality risk; 26% agreed or strongly agreed, M=3.51, SD=2.08, t(177)=2.52, p<0.05).

**Figure 4:**
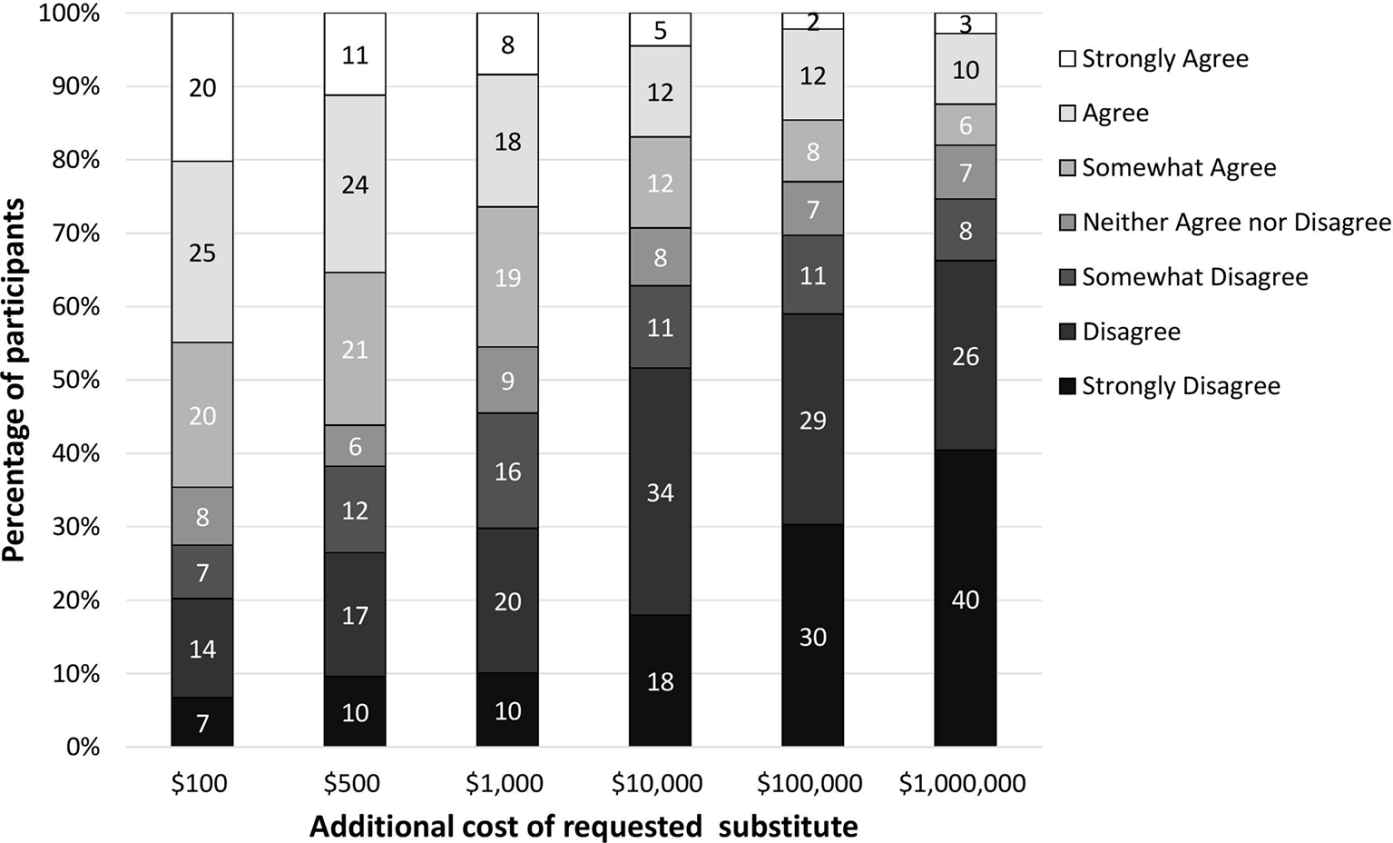
Level of agreement to provide alternative treatments of increased expense.

Many participants agreed or strongly agreed with providing treatment that added $100 (45%) or $500 (35%) to the original $400 treatment. Respondents were less inclined to agree as the cost of the substitute treatment increased (*F*(5,885)=121.79, p<0.001) (figure 4). A large proportion of respondents disagreed or strongly disagreed with providing substitute treatments that cost an additional $100 000 (59%) or $1 000 000 (66%).

Next, we looked at how participants’ support for substitute treatments varied based on whether the reason behind the parents’ request was religious or ethical (non-religious). Results showed that, overall, participants were more inclined to allow parental requests when given a religious reason; though the difference was only statistically significant for the three substitute treatment requests that had an increased risk of death (small risk, t(176)=2.32, p=0.02; medium risk, t(176)=2.01, p=0.05; large risk, t(176)=2.39, p=0.02) (Appendix D).

### Section 3

**Figure 5:**
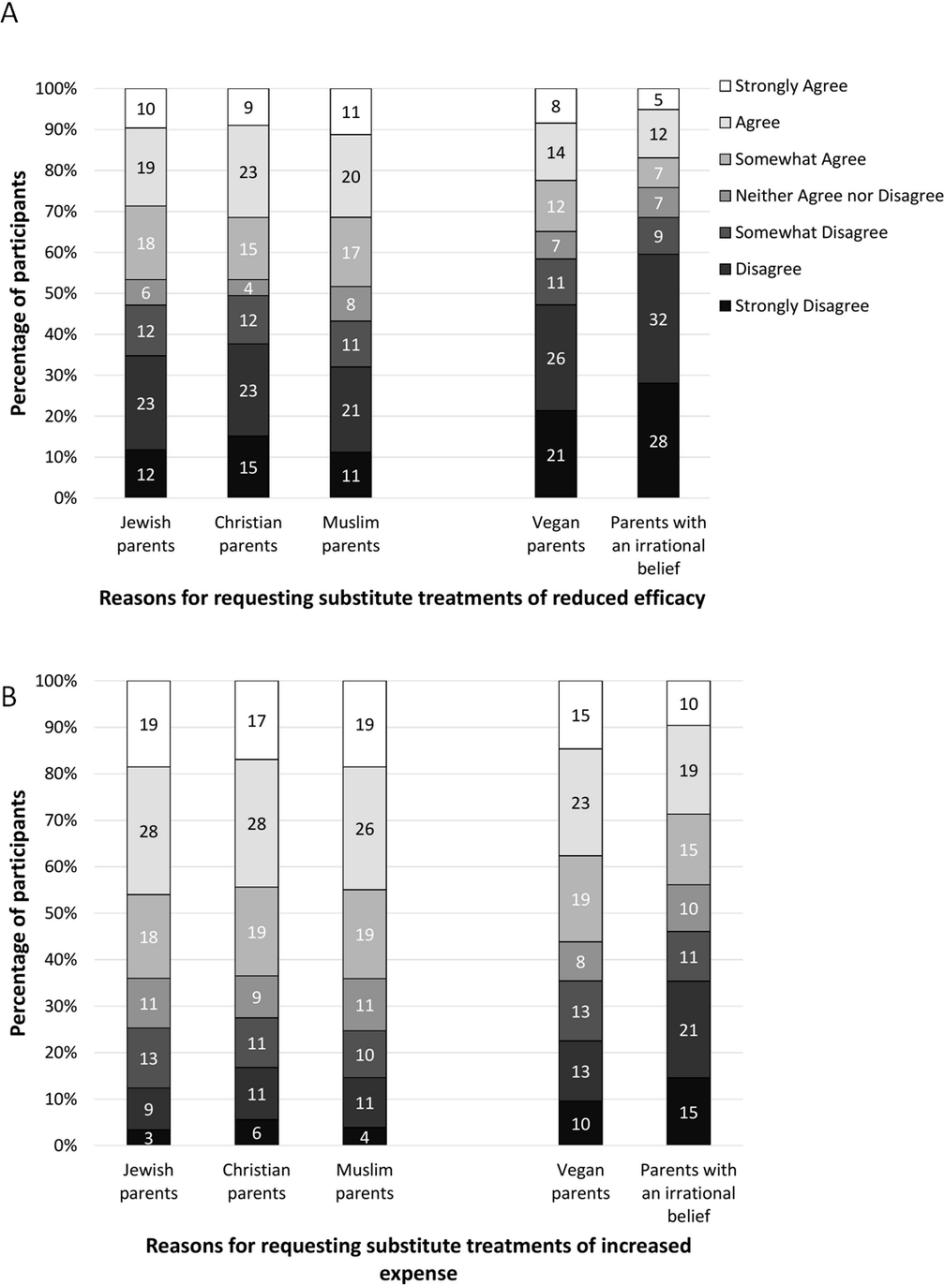
Level of agreement to provide substitute treatments of reduced efficacy (**A**) and increased expense (**B**) when requested for varying reasons.

In a fully within-subjects design, respondents were presented with three religious and two non-religious reasons for parents requesting either a less effective substitute treatment or a more expensive treatment. A repeated measures analysis of variance revealed the main effects of both the reason for the request (*F*(4,708)=50.46, p<0.001) and the type of treatment (*F*(1,177)=52.63, p<0.001) on how much participants endorsed the substitute treatment, though there was no interaction effect between the two (*F*(4,708)=1.23, p=0.30). That is, participants were more willing to agree with a request for substitute treatment when this was based on religious (vs non-religious) reasons (see figure 5), and when the substitute treatment was more expensive (vs having a higher side effect of death). Looking only at the category of non-religious reasons, participants were more willing to provide treatment at the request of vegan parents compared with parents with an irrational belief for both the less effective substitute (t(177)=4.20, p<0.001) and for the more expensive substitute (t(177)=4.72, p<0.001) (see figure 5A,B and Appendices E and F).

### Section 4

Finally, we looked at how participants’ judged requests for treatments based on religious versus non-religious reasons in the two contexts of prolonging futile treatment and refusing a blood transfusion. Participants did not differ in their endorsement of prolonging futile treatment based on whether this request was based on religious (M=5.01, SD=1.66) or non-religious (M=4.99, SD=1.66) reasons (t(177)=0.46, p=0.64). However, participants were less willing to agree with a request to refuse a blood transfusion for religious reasons (M=4.08, SD=2.11) compared with non-religious reasons (M=4.35, SD=2.05) (t(177)=3.12, p=0.002) (Appendix G).

### Section 5

There was no association between participants’ responses to the survey and their age, gender, employment status, ethnicity, whether they had children or their response to the Speciesism Scale. A weak association was found in results between responses and levels of education. Participants with a higher level of education indicated lower levels of agreement to provide substitute requested treatment associated with a small increased risk of death or to provide treatment for vegan parents.

Overall, highly religious participants had higher agreement scores than not religious participants (ie, were more likely to agree to provide treatment substitutes) for all scenarios except for the blood transfusion refusal scenario, but these differences were not statistically significant^ii^ (Appendix H).

## Discussion

To our knowledge, this is the first study to gauge the attitudes of the general public towards parental refusal of treatment or requests for substitute treatment on behalf of their children. Participants were presented with substitute treatments of varying efficacy and cost, and different reasons for parental requests. Many of those surveyed were prepared to support parental refusal of treatment or request for less effective treatment even where this would increase a child’s risk of dying or of disability. Participants were more likely to refuse requests for substitutes as the efficacy of the substitute fell, or its cost increased. Respondents were more inclined to agree if provided with a religious reason for parents’ request compared with a non-religious reason. Responses were not influenced by age, gender, employment status, whether respondents had children, dietary preference or ethnicity. We defined a ‘threshold’ for approval as the point at which the majority of respondents agreed to a parental request. In our survey, requests for substitute treatments with a small increased risk of death (<5%) were supported by a majority. The cost threshold sat at an additional cost of $100–$500.

The findings of the study were consistent with our hypotheses. As the absolute risk of death associated with substitute treatments increased, the percentage of respondents who strongly disagreed or (simply) disagreed to allow the request increased significantly. This finding fits with an assessment that the balance of interests tips in the child’s favour as the risk of harm or death becomes greater; consequently it becomes more reasonable to over-rule parental requests.

Participants were less likely to allow a substitute that increased a risk of disability than one that increased a risk of pain, reflecting the reasonable assessment that long-term disability is a more serious harm.

Overall, although some differences were statistically significant, respondents’ opinions in our survey were divided between agreement and disagreement for all questions. Even for the most serious risks (disability and a large increased risk of death), a relatively large proportion of the cohort supported parents’ request, showing a striking willingness to allow parents to make decisions that risked harm to their children. Nearly a third of all participants agreed or strongly agreed to a parental refusal of treatment that would result in a 15% increase in the absolute risk of mortality. This is notably different from norms in clinical practice. In a survey of 957 American oncologists,^16^ over 80% found it unacceptable to allow parents to refuse chemotherapy for children with moderate to good prognosis.

Paradoxically, there was higher agreement to allow parents to refuse treatment (30% absolute mortality risk) than to provide a substitute, but relatively ineffective treatment (29% absolute mortality risk). Perhaps participants intuitively distinguished between an act and an omission, and were less willing to allow parents to *request* a medication (a positive right) than to *refuse* treatment (a negative right) which is a more familiar aspect of clinical practice.^17^ Alternatively, it is possible that participants simply did not make a logical connection between the two questions, or that responses were impacted by respondents’ difficulties in understanding statistical risk^18 19^

A clearer relationship was evident for the questions relating to expense than efficacy; there was a strong negative correlation between additional cost requested and agreement. One possible explanation for this is that intervals in cost may be easier to understand and more intuitively comparable than the intervals in efficacy. A small proportion of respondents were prepared to support parental requests for treatment substitutes even at considerable excessive cost.

As anticipated, the results showed religiously motivated requests to be met with higher agreement. This apparent bias towards religion could be because religion has a higher status in society than other personal beliefs (such as dietary preferences for ethical reasons), reflected in the legal or constitutional protection of religion in the USA,^20^ Australia,^21^ Europe^22^ and many other countries. However, a religiously motivated parental refusal of blood transfusion had a lower agreement score than a non-religion-based refusal (this was true of all participants, irrespective of religiosity). It is possible that participants had pre-existing negative judgements of Jehovah’s Witnesses, or believed the risk of blood-borne infections (the non-religious reason) to be more serious or plausible.

The religiosity of the respondents was not significantly correlated with higher agreement to religion-based requests. This differed from other studies^23 24^ that have shown a physician’s religious preferences to influence her decision making. A survey of 446 US physicians^25^ showed that physicians with high religiosity were substantially more likely to refuse a request they considered morally wrong. However, many of these studies are about end-of-life care, a matter intrinsically linked to religious morality, where the decision may more directly relate to the physician’s personal religious beliefs.

### Limitations

We deliberately sought the views of the general community, however, it is possible that parents of children with medical problems, or health professionals would have a different response to scenarios. Participants were also asked to assume the role of the doctor and this artificial instruction may have influenced their responses.^26^

As is the case with all online experimentation, we could not control the participants’ experimental setting, and ecological validity cannot be assured.^27^ All MTurk experiments have the potential for respondents to give satisficing responses, or for software programs to automatically complete HITs.^10^ To counter this, an attention check was added and inadequate responses removed. Our sample had sufficient statistical power to detect a modest effect in the primary analysis. However, it was not powered to detect small subgroup differences, and with a larger sample, the participants could have been stratified by religious affiliation or religiosity to compare the results more thoroughly. All participants were from the USA and thus responses may reflect local, cultural and religious values, particularly their opinion of the role of a public healthcare system and how it ought to allocate its resources. While MTurk respondents have previously been shown to be broadly representative of the US population, our survey respondents had a relatively high proportion of educated, female respondents, and results may not be generalisable to other populations and settings. Finally, our quantitative study was not able to explore the reason behind the respondents’ choices.

## Part 2: Ethical analysis

Our data show how some members of the general public respond to hypothetical cases of parental requests for substitute medical treatment, but how *should* we respond? A detailed theoretical analysis is beyond the scope of the paper, however, as a starting point we draw on a recently published systematic review.^9^ We summarise several existing ethical frameworks that have been used to decide about parents’ medical decision making for their child and apply them to the clinical scenario summarised at the start of this paper (box 1). We compare empirical findings to theory, with the goal of achieving coherence between ethical principles and data gathered of people’s beliefs and intuitions, working towards wide reflective equilibrium.^28^ We will propose a preliminary framework building on this analysis.

Several normative models have previously been proposed to assess whether or not it is justified for a physician to over-ride parents’ medical decision making for their child. McDougall and Notini, in a recent paper, identified nine different frameworks.^9^ Table 1 summarises a subset of the most relevant of these frameworks and includes another, more recent, one which deals explicitly with parental requests as well as refusals.^29^

**Table 1:** Five ethical frameworks for assessing the permissibility of parental choices around medical care (modified and edited from McDougall and Notini^9^ and Gillam^29^)

Ethical framework	Summary
*Best Interests* Kopelman^8^ Buchanan and Brock^49^	‘Acting to promote maximally the good of the individual (the child)’ • The state can over-ride the parents’ authority if the child has suffered or is in danger of suffering serious harm • Once that threshold is met, the state must decide on the course of action considering the child’s best interests
*Harm Principle* Diekema^7^	Health professionals can seek state intervention if: • The parents’ decision significantly increases the risk of harm • The harm is imminent • The refused intervention is necessary to prevent the harm • The refused intervention is of proven efficacy • The projected benefit to burden ratio of the refused intervention is significantly more favourable compared with that of the parents’ preferred option • No alternative would prevent harm and be more acceptable to the parents • Most parents would agree that state intervention was reasonable
*The Not Unreasonable Standard* Rhodes and Holzman^31^	Assess the appropriateness of the decision makers and assess the appropriateness of the decision itself • *Centre core judgement*: one that is universally made and cannot be reasonably rejected • *Second domain judgement*: one that is prioritised differently by reasonable people • *Third domain judgement*: one that can be reasonably rejected An idiosyncratic reason can only be accepted when a patient makes a decision for themselves, not when a surrogate makes a decision (eg, parents). ‘Only decisions based on universal reasons are acceptable for surrogate refusal of highly beneficial treatment.’
*Balance of Cost and Benefits* DeMarco *et al* 32	An economic theory which balances: • Cost of treatment for the patient • Benefit of treatment for the patient • Costs borne by others as a result of the treatment
*Zone of Parental Discretion* Gillam and Kilham *et al* 29 50	A practical tool which denotes an ‘ethically and legally protected space’ in which parents are allowed to make decisions for their children. It considers: • The Harm Principle (see above) • The burdensomeness of the intervention Compares the expected harms of acceding to the parents’ wishes with the harms involved in over-riding the parents’ wishes

Applying these frameworks to the case example yields varying outcomes (table 2). A strict interpretation of the Best Interests Standard would mean denying any parental request for a less effective treatment.^30^ It is not clear whether this standard would permit more expensive (but equally effective) substitutes. However, other frameworks did not yield a clear answer to whether parents should be permitted to choose suboptimal substitute treatment. Although Rhodes and Holzman’s ‘Not Unreasonable Standard’^31^ classifies beliefs held by only subgroups of the population (such as religion) as third domain judgements which can be reasonably rejected (table 1), the authors argue that parents’ decisions should only be over-ruled if the prognosis is poor; and it is unclear how poor that prognosis must be. DeMarco *et al*’s ‘Balance of Costs and Benefits’ framework^32^ does not state how one ought to weigh the costs and benefits of treatment to come to a decision. Diekema’s Harm Principle and Gillam’s Zone of Parental Discretion appear to map most closely onto current clinical decision making^iii^ but do not clearly state what constitutes a ‘significant risk’ or a ‘serious harm’ and thus fail to yield a clear answer.

**Table 2:** The solutions provided by the theoretical frameworks for over-riding parental decisions, applied to a request for a substitute form of surfactant replacement therapy

Framework	Status of request
Best Interests Standard	Denied
Harm Principle	Unclear
Not Unreasonable Standard	Unclear
Balance of Costs and Benefits	Unclear
Zone of Parental Discretion	Unclear

Where should we draw the line on parental requests for substitute medical treatment? There is no clear theoretical answer to this question. Although empirical surveys do not settle the question either, the intuitions of the general public provide a potential starting point for achieving coherence between intuitions and theory using the reflective equilibrium.^28^ Empirical evidence that is inconsistent with one’s position should encourage further analysis, and re-evaluation of previous reasoning. For example, if the general public believe that a 5% risk of death is acceptable, yet an ethicist may only accept 0.01%, this says that the general public value parental autonomy more than the ethicist. The ethicist ought to assess the value of parental autonomy and consider what arguments could justify raising the threshold above 0.01%, even though those arguments might not support going all the way to 5%. That is, the empirical evidence causes us to reconsider arguments and reasons, and perhaps choose another point within a justifiable options space.

There are some apparent inconsistencies between the ethical frameworks and the results of our empirical survey. First, a significant proportion of our cohort judged it acceptable for parents to make decisions that were associated with serious risks for the child. Even if the frameworks are unclear about requests for less effective treatments, it seems that a 15% absolute increase in the risk of death for a child (as was the case with an outright refusal of treatment) represents a significant risk of serious harm, and would not be supported by any of the models. Second, in our survey, there was a clear bias towards religious reasons and against non-religious ethical beliefs such as veganism. This appears to demonstrate discrimination against parents with sincere non-religious beliefs. However, ethicists have argued that patient or parent’s religion should not be considered a relevant difference when considering otherwise alike cases.^29 33^ Of the models described in table 1, only the ‘Not unreasonable framework’ makes a clear difference based on the *reason* behind parental requests. In that model, central core judgements provide an acceptable reason for parents to make requests for suboptimal treatment. In contrast to our survey findings, that framework suggests that a secular preference for non-animal-based treatment should be given more weight than a religious reason.

A majority of the general public surveyed were comfortable allowing parents to choose treatment that would result in up to a <5% increase in the absolute risk of death. If this were used as the threshold for over-ruling parental decisions, 20 parents (or fewer) would need to be over-ruled to prevent one death. Five per cent represents a statistical threshold often used in scientific studies for judging low probability of error, as well as the threshold that some have argued should be applied to judgements ‘beyond reasonable doubt’ in criminal prosecutions,^34^ however, some may regard 5% as still too high a level of risk to impose on a child for the sake of parental values. Other risks that children are subjected to by their parents, such as passive smoking or unsafe driving, would not increase risk of death by as much. The risk of death from driving in a year is approximately 1/10 000,^35^ and is universally regarded as an acceptable risk for parents to impose on their children. Perhaps the threshold should be set at 1%, 0.1% or 0.01%? We cannot definitively answer that question here.

In fact, decision theory would suggest that there are two variables in setting a threshold level of harm: the *severity* of harm and the *probability* of this harm occurring.^36^ It may be reasonable to allow a higher probability where the severity of the harm is less. If an expected harm approach (drawing on decision-theoretic consequentialism) is the correct approach to medical uncertainty, we could extrapolate from the empirical results to create a set of potential harm thresholds to decide when a risk should be tolerated (table 3).

**Table 3:** Potential harm thresholds for allowing parents to choose less effective treatment

Severity of harm	Example harms	Example case	Acceptable probability of risk eventuating
Serious	Death Long-term disability	Refusing a vitamin K injection for a newborn could lead to clotting and has an increased risk of death^30^	Small <5%
Medium	Painful procedure Additional surgery Short-term illness	Refusing a complete surgical repair of a broken leg (because it requires a blood transfusion) and instead opting for an incomplete repair, increasing the need for additional surgeries and the risk of surgical complications^51^	Medium <15%
Small	Somewhat painful procedure Slightly increased length of illness	Refusing antibiotics for an ear infection may lengthen the duration of the illness^52^	Large <50%
Trivial	Very short-lasting pain	Ear piercing causes mild pain that quickly resolves^53^	Certain 100%

What cost would be acceptable for parental choice? The results of this study demonstrate a willingness to provide treatment up to $500 more expensive at parental request. The plausibility of this threshold will depend on the availability of resources within a public health system. Very wealthy countries may be able to afford a higher amount, while countries that struggle to afford basic health services might decide to prohibit any parental choices that would be more costly. In practice, the $500 threshold would potentially rule out many substitute treatments, since small differences in the efficacy of treatment could lead to additional medical costs including additional bed days or need for future medical interventions.

Wherever we set the threshold for more expensive treatment, one option will be to permit parents to pay the excess cost of their preferred treatment. This would largely remove the ethical reason to decline parental request (assuming the treatment was not more harmful), and we would propose this should be allowed. However, this option would conflict with strict egalitarian policies like those adopted in the National Health Service.^37^

How should doctors take into account the reasons behind parental requests? One option would be to vary thresholds for cost or harm depending on the reason. On this basis it may be ethical to allow a more harmful or more costly choice where parents are motivated by a particularly weighty reason. However, such variable thresholds might well be anticipated to lead to significant inconsistency in practice, as well as bias against certain groups due to physicians’ variable interpretation of the weightiness of reasons. On the other hand, it appears unacceptable to allow parents to choose suboptimal treatment for any reason whatsoever: parents should not be able to choose more harmful or more costly treatments for trivial or irrational reasons. In such cases, the cost borne by the child will outweigh the benefit gained by the parents, if any. (The latter might yield genuine questions about the competence of parents to make decisions for their child.)

**Figure 6:**
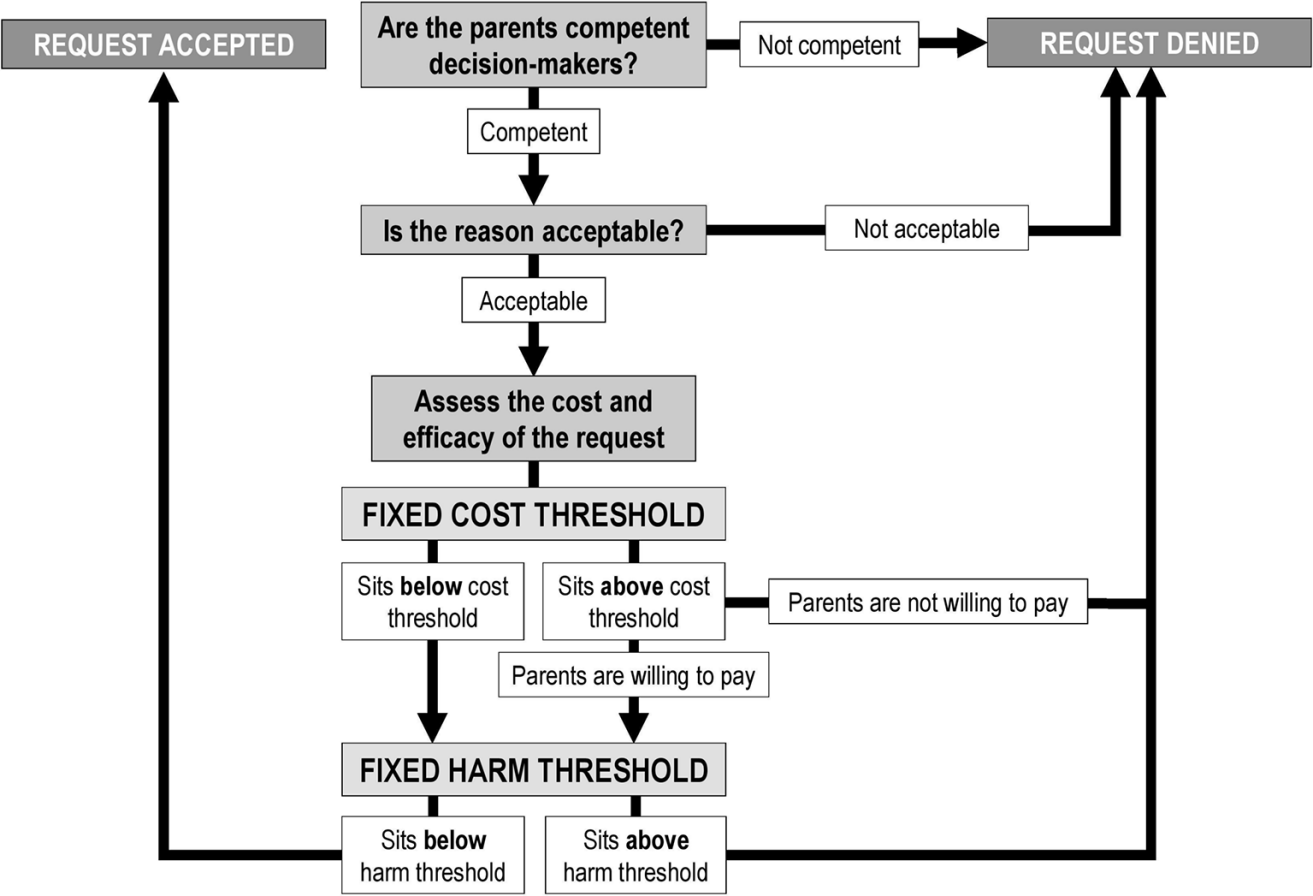
Reasonable-Choice Threshold Model for parental choice around treatment.

The Reasonable-Choice Threshold Model (figure 6) combines the above ethical conclusions into a model for arbitrating parental decisions around refusal of treatment or requests for suboptimal treatment.^iv^

In this model, the doctor should first assess the competency of the decision maker, next consider the acceptability of their reason and finally, if the reason is acceptable, the request should be subjected to fixed cost and harm thresholds. Which reasons are acceptable? Intuitively, those that are clearly articulated, motivated by genuine concern for the child’s well-being, persistent and represent core values associated with long-standing tradition would be deemed acceptable by many, this may include religious values. However, secular reasons may also potentially fulfil these criteria, and the model should not discriminate between religious and non-religious adherents. A full articulation of the distinction between acceptable and unacceptable reasons requires further research and lies beyond the scope of this paper.

Applying the Reasonable-Choice Threshold Model to our case from the beginning of this paper (box 1) yields the conclusion that the parents’ request should arguably be permitted. We did not provide information about parents’ competence to decide, but this may be assumed for the sake of argument. Is the reason acceptable? Islam is a well-established religion with many millions of followers practising the same customs for thousands of years. Respect for the large number of adherents and longevity of the tradition would arguably make such a reason acceptable. The request must then be subjected to the cost and harm thresholds. There are relatively little contemporary data on the relevant costs of surfactant therapy, however, one study comparing the costs of acquisition, administration and adverse events found beractant (bovine) to be $493 more expensive than poractant alfa (porcine)^38^; on the model above it sits just below the cost threshold. Is the level of harm acceptable? Focusing just on the most serious risk—that of death, the absolute risk reduction in mortality associated with poractant alfa is 6.4% and beractant is 10.9%,^39^ which is about a 4.5% absolute increase and sits below the harm threshold as set out in table 3.

## Conclusions

This study has explored the boundaries of parental choice in medical decision making for children. Expanding medical possibilities, expanding access to information and increasing diversity of values in society are likely to lead to more situations where parents request treatment that differs from those recommended by health professionals. It will be important to determine when such requests should be accepted, and when they should not.

Our study brings together empirical research and ethical analysis. As noted above, the empirical findings may not be generalisable to other populations, and a repeat survey in a group with different values or norms might yield significantly different thresholds. Endorsement of a particular view by the general public does not necessarily mean this is the ethically correct approach. Where a process of wide reflective equilibrium identifies a conflict between ethical theory and the intuitions of the general public, it may be appropriate either to reconsider or modify the theory, or to reject the intuitions. However, it is not always clear which course to take.

Our survey sample appeared to have intuitions which were most consistent with the Harm Model or Zone of Parental Discretion. They were inconsistent with the Best Interests Standard which officially governs medical ethics and practice.

We have outlined one potential model for arbitrating decisions about suboptimal treatment, and drawn on our empirical work to suggest some potential harm and cost thresholds as a starting point for debate. An expected harm approach which considers both magnitude and probability of harm is a promising approach which appears consistent with public intuition. Further empirical study, including qualitative research, is necessary to help test our proposed model against other medical decisions, and assess the views of stakeholders, including medical professionals, about it. Further ethical research will also be crucial, in particular to address the difficult question of the acceptability of different reasons underlying parental choice.

## Footnotes

i Caviola, L., Everett, J. A. C., Mannino, A., & Faber, N. S. (2016), manuscript in preparation.

ii The middle category,‘Religious' (N=62), was not included in this analysis.

iii This is not to say that current clinical practice is necessarily correct, however it is important to compare suggestions offered by theoretical frameworks to existing guidelines and practice.

iv The model is derived from analysis of the ethical considerations at stake in decisions about substitute treatment, as well as review of theoretical models previously published. This generated a set of normative conclusions that form the foundation of the model: Parents’ requests for suboptimal treatments should be rejected if the parents are not competent to decide, or the reasons appear trivial or irrational; requests for suboptimal treatment may be permissible if the additional cost to the public health system is not excessive, and if the harm caused to the child is not excessive.
