# Supplementary material for: Settling for second best: when should doctors agree to parental demands for suboptimal medical treatment?
Source: J Med Ethics. 2017 Sep 25;43(12):831–40. doi: 10.1136/medethics-2016-103461 (PMC5827708; doi:10.1136/medethics-2016-103461)
Supplement: Supplementary file [file medethics-2016-103461supp001.pdf]

APPENDIX

Appendix A. Survey

SECTION ONE:

A very premature baby has just been born. The baby is seriously ill and in intensive care with difficulty breathing. Babies with this illness have approximately 1 in 3 chance (33%) of dying.

There is a medicine available ("Medicine A") to treat premature babies with this sort of illness. It reduces the baby's chance of dying to 1 in 6 (17%). If 100 infants are treated with medicine A, 15 infants will have their lives saved.

[The parents of this child have an objection against this treatment. They tell you that the reason they are objecting to Medicine A is because it is forbidden by their **religion**. They want what's best for their child but cannot permit the use of this medication because they believe it would prevent the child's eternal salvation.]

// OR ***//***

[The parents of this child have an objection against this treatment. They tell you that the reason they are objecting to Medicine A is because the main ingredient of the drug is derived from an animal and they consider it ethically wrong to use animal products for human benefit.]

In the following scenarios, imagine yourself to be in the position of the **doctor**. You will be asked whether you agree or disagree with allowing the parents' request.

How much do you agree with the following statement?

You should permit the parents to refuse the medicine with the result that their child will be left untreated?

Strongly Disagree	Disagree	Somewhat Disagree	Neither Agree nor Disagree	Somewhat Agree	Agree	Strongly Agree
○	○	○	○	○	○	○

There is a different form of Medicine A available that the parents will accept ("Medicine B"). However Medicine B is **less effective** than Medicine A. Medicine A and B are the **same cost**.

There is no difference in the risk of complications but Medicine B has a **higher risk of death**. If 100 infants are treated with Medicine B rather than A, 4 less infants will survive (**only 11 will have their lives saved**). How much do you agree with the following statement?

You should allow the parents to choose this different treatment for their child.

Strongly Disagree	Disagree	Somewhat Disagree	Neither Agree nor Disagree	Somewhat Agree	Agree	Strongly Agree
○	○	○	○	○	○	○

There is no difference in the risk of death, but Medicine B has a 10% higher risk of bleeding in the brain. Bleeding in the brain could result in **physical and mental disability** for the child. How much do you agree with the following statement?

You should allow the parents to choose this different treatment for their child.

Strongly Disagree	Disagree	Somewhat Disagree	Neither Agree nor Disagree	Somewhat Agree	Agree	Strongly Agree
○	○	○	○	○	○	○

There is no difference in the risk of death, but Medicine B has a 10% higher risk of air leak around the lung. If the child develops an air leak they will need a **painful** procedure to drain the air. How much do you agree with the following statement?

You should allow the parents to choose this different treatment for their child.

Strongly Disagree	Disagree	Somewhat Disagree	Neither Agree nor Disagree	Somewhat Agree	Agree	Strongly Agree
○	○	○	○	○	○	○

There is no difference in the risk of complications, but Medicine B has a **higher risk of death**. If 100 infants are treated with Medicine B rather than A, 14 less infants will survive (**only 1 will have their life saved**). How much do you agree with the following statement?

You should allow the parents to choose this different treatment for their child.

Strongly Disagree	Disagree	Somewhat Disagree	Neither Agree nor Disagree	Somewhat Agree	Agree	Strongly Agree
○	○	○	○	○	○	○

There is no difference in the risk of complications, but Medicine B has a **higher risk of death**. If 100 infants are treated with Medicine B rather than A, 9 less infants will survive (**only 6 will have their lives saved**). How much do you agree with the following statement?

You should allow the parents to choose this different treatment for their child.

Strongly Disagree	Disagree	Somewhat Disagree	Neither Agree nor Disagree	Somewhat Agree	Agree	Strongly Agree
○	○	○	○	○	○	○

SECTION TWO:

In a public healthcare system, there is a finite and often limited amount of resources. Extra money spent on one patient may mean that treatment is not available for another patient.

There parents are low income earners and qualify for Medicaid; a government funded program that provides health coverage to those who are strugglien financially. The treatment of this premature baby is being publicly funded. Medicine A costs $USD400 per treatment.

For the following scenarios, assume that Medicine B will produce the **same health outcomes** for an infants as Medicine A. However, Medicine B is **more expensive** than Medicine A.

Medicine B costs an **extra $100** (this is the cost of giving 10 patients a course of antibiotics). How much do you agree with the following statement?

You should allow the parents to choose this different treatment for their child.

Strongly Disagree	Disagree	Somewhat Disagree	Neither Agree nor Disagree	Somewhat Agree	Agree	Strongly Agree
○	○	○	○	○	○	○

Medicine B costs an **extra $500** (this is the cost of giving 16 patients a yearly flu shot). How much do you agree with the following statement?

You should allow the parents to choose this different treatment for their child.

Strongly Disagree	Disagree	Somewhat Disagree	Neither Agree nor Disagree	Somewhat Agree	Agree	Strongly Agree
○	○	○	○	○	○	○

Medicine B costs an **extra $1000** (this is the cost of performing 4 abdominal ultrasounds). How much do you agree with the following statement?

You should allow the parents to choose this different treatment for their child.

Strongly Disagree	Disagree	Somewhat Disagree	Neither Agree nor Disagree	Somewhat Agree	Agree	Strongly Agree
○	○	○	○	○	○	○

Medicine B costs an **extra $10,000** (this is the cost of treatment for a patient with leukaemia for 1 month). How much do you agree with the following statement?

You should allow the parents to choose this different treatment for their child.

Strongly Disagree	Disagree	Somewhat Disagree	Neither Agree nor Disagree	Somewhat Agree	Agree	Strongly Agree
○	○	○	○	○	○	○

Medicine B costs an **extra $100,000** (this is the cost of performing a complete hip replacement surgery on 5 patients). How much do you agree with the following statement?

You should allow the parents to choose this different treatment for their child.

Strongly Disagree	Disagree	Somewhat Disagree	Neither Agree nor Disagree	Somewhat Agree	Agree	Strongly Agree
○	○	○	○	○	○	○

Medicine B costs an **extra $1,000,000** (this is the cost of giving a kidney transplant to 4 patients). How much do you agree with the following statement?

You should allow the parents to choose this different treatment for their child.

Strongly Disagree	Disagree	Somewhat Disagree	Neither Agree nor Disagree	Somewhat Agree	Agree	Strongly Agree
○	○	○	○	○	○	○

Medicine B costs an **extra $1,000,000** (this is the cost of giving a kidney transplant to 4 patients). This is a test question to make sure you are paying attention.

Please leave this question blank.

Strongly Disagree	Disagree	Somewhat Disagree	Neither Agree nor Disagree	Somewhat Agree	Agree	Strongly Agree
○	○	○	○	○	○	○

<u>SECTION THREE:</u>

Another couple have had a premature baby who is also in need of Medicine A. They too object to the use of this medication.

The parents have approached you (the doctor) with their request for an alternative treatment. You sit down with them to have a discussion as to **why** they are objecting to this treatment.

You will be asked how much you agree with the following statements.

The parents object to Medicine A because it is derived from pork. They are **Jewish** and believe the use of a pig-derived drug is unacceptable.

The alternative medicine they are requesting has a **higher chance of death** than Medicine A. How much do you agree with the following statement?

You should allow the parents to choose this alternative treatment for their child.

Strongly Disagree	Disagree	Somewhat Disagree	Neither Agree nor Disagree	Somewhat Agree	Agree	Strongly Agree
○	○	○	○	○	○	○

The alternative medicine they are requesting is **double the cost** of Medicine A. How much do you agree with the following statement?

You should allow the parents to choose this alternative treatment for their child.

Strongly Disagree	Disagree	Somewhat Disagree	Neither Agree nor Disagree	Somewhat Agree	Agree	Strongly Agree
○	○	○	○	○	○	○

The parents object to Medicine A because it is derived from pork and they are **vegans**.

The alternative medicine they are requesting has a **higher chance of death** than Medicine A. How much do you agree with the following statement?

You should allow the parents to choose this alternative treatment for their child.

Strongly Disagree	Disagree	Somewhat Disagree	Neither Agree nor Disagree	Somewhat Agree	Agree	Strongly Agree
○	○	○	○	○	○	○

The alternative medicine they are requesting is **double the cost** of Medicine A. How much do you agree with the following statement?

You should allow the parents to choose this alternative treatment for their child.

Strongly Disagree	Disagree	Somewhat Disagree	Neither Agree nor Disagree	Somewhat Agree	Agree	Strongly Agree
○	○	○	○	○	○	○

The parents object to Medicine A because it is derived from pork and the mother **believes she is allergic to pork**. She fears the baby will be too. As a doctor you know that this is not a realistic possibility.

The alternative medicine they are requesting has a **higher chance of death** than Medicine A. How much do you agree with the following statement?

You should allow the parents to choose this alternative treatment for their child.

Strongly Disagree	Disagree	Somewhat Disagree	Neither Agree nor Disagree	Somewhat Agree	Agree	Strongly Agree
○	○	○	○	○	○	○

The alternative medicine they are requesting is **double the cost** of Medicine A. How much do you agree with the following statement?

You should allow the parents to choose this alternative treatment for their child.

Strongly Disagree	Disagree	Somewhat Disagree	Neither Agree nor Disagree	Somewhat Agree	Agree	Strongly Agree
○	○	○	○	○	○	○

The parents object to Medicine A because it is a stem cell treatment derived from the tissue of aborted fetuses. They are **Christian** and believe that the use of a drug made from the tissue of aborted fetuses is unacceptable.

The alternative medicine they are requesting has a **higher chance of death** than Medicine A. How much do you agree with the following statement?

You should allow the parents to choose this alternative treatment for their child.

Strongly Disagree	Disagree	Somewhat Disagree	Neither Agree nor Disagree	Somewhat Agree	Agree	Strongly Agree
○	○	○	○	○	○	○

The alternative medicine they are requesting is **double the cost** of Medicine A. How much do you agree with the following statement?

You should allow the parents to choose this alternative treatment for their child.

Strongly Disagree	Disagree	Somewhat Disagree	Neither Agree nor Disagree	Somewhat Agree	Agree	Strongly Agree
○	○	○	○	○	○	○

The parents object to Medicine A because it is derived from pork. They are **Muslim** and believe the use of a pig-derived drug is unacceptable.

The alternative medicine they are requesting has a **higher chance of death** than Medicine A. How much do you agree with the following statement?

You should allow the parents to choose this alternative treatment for their child.

Strongly Disagree	Disagree	Somewhat Disagree	Neither Agree nor Disagree	Somewhat Agree	Agree	Strongly Agree
○	○	○	○	○	○	○

The alternative medicine they are requesting is **double the cost** of Medicine A. How much do you agree with the following statement?

You should allow the parents to choose this alternative treatment for their child.

Strongly Disagree	Disagree	Somewhat Disagree	Neither Agree nor Disagree	Somewhat Agree	Agree	Strongly Agree
○	○	○	○	○	○	○

SECTION FOUR:

In the following scenarios we would like you to again imagine that you are a doctor. You are caring for a child whose parents disagree with the treatment that you have advised.

A premature baby is extremely ill and only has a 1 in 1000 chance of survival. You (the doctor) and your medical team believe it is time to stop treatment, as the chance of survival is very low and the treatment is causing the child to suffer.

The parents request that you **continue providing the treatment** for 1 month. They are devoutly **religious**. Although they understand that there is only a very small chance of recovery, they are praying very hard and believe that a miracle will occur. How much do you agree with the following statement?

You should continue to provide the treatment

Strongly Disagree	Disagree	Somewhat Disagree	Neither Agree nor Disagree	Somewhat Agree	Agree	Strongly Agree
○	○	○	○	○	○	○

The parents request that you continue providing the treatment for 1 month. They are **not religious**. Although they understand that there is only a very small chance of recovery, they hope that their child will defy the odds. How much do you agree with the following statement?

You should continue to provide the treatment

Strongly Disagree	Disagree	Somewhat Disagree	Neither Agree nor Disagree	Somewhat Agree	Agree	Strongly Agree
○	○	○	○	○	○	○

A premature baby is very ill and has a very low blood count (they are severely anaemic). The medical team believe that the baby **requires a blood transfusion** which will improve their chance of survival.

The parents do not wish their child to have a blood transfusions because they are **Jehovah's witnesses**. How much do you agree with the following statement?

You should transfuse the baby against the parents' wishes

Strongly Disagree	Disagree	Somewhat Disagree	Neither Agree nor Disagree	Somewhat Agree	Agree	Strongly Agree
○	○	○	○	○	○	○

The parents do not wish their child to have a blood transfusion because they have read that blood transfusions have a small risk of **transmitting a serious infection** like hepatitis. How much do you agree with the following statement?

You should transfuse the baby against the parents' wishes

Strongly Disagree	Disagree	Somewhat Disagree	Neither Agree nor Disagree	Somewhat Agree	Agree	Strongly Agree
○	○	○	○	○	○	○

<u>SECTION FIVE:</u>

Please answer the following questions about yourself. This helps us to understand survey responses

Please note that all the information you provide (for the entire questionnaire) is completely anonymous.

1. What is your age?
    a. 18 – 24 years
    b. 25 – 34 years
    c. 35 – 44 years
    d. 45 – 54 years
    e. 55 – 64 years
    f. 65+ years

2. How do you describe your gender?
    a. Male
    b. Female
    c. Other

3. What is your highest level of educational qualification? (If currently enrolled, highest degree received)
    a. No formal qualification
    b. Did not complete Secondary School
    c. Graduated from Secondary School (includes equivalency)
    d. Vocational qualification (Apprenticeship, etc.)
    e. Non-tertiary qualification (diploma, associate degree, etc.)
    f. Undergraduate tertiary qualification (Bachelors)
    g. Postgraduate tertiary qualification (Honours, Masters, PhD)

4. Are you currently….?
    a. Employed
    b. A student
    c. A homemaker
    d. Out of work
    e. Unable to work

5. Do you have any children?
    a. Yes
    b. No

6. What country do you live in? [Drop down box]

7. What is your ethnic background?
    a. Asian
    b. Australasian
    c. European
    d. North American
    e. South American
    f. African

8. Do you regard yourself as belonging to any particular religion?
    a. Yes
    b. No, I'm an atheist
    c. Not sure
    d. I'd rather not say

9. If YES to question 6, which religion to you belong to?
    a. Christianity
    b. Buddhism
    c. Hinduism
    d. Islam
    e. Judaism
    f. Other
    g. I'd rather not say
    h. I selected "No" or "Not sure" for the previous question

10. How often do you think about religious issues?
    a. Never
    b. Rarely
    c. Occasionally
    d. Often
    e. Very Often

11. To what extent do you believe that God or something divine exists?
    a. Not at all
    b. Not very much
    c. Moderately
    d. Quite a bit
    e. Very much so

12. How often do you take part in religious services?
    a. Never
    b. Rarely
    c. Occasionally
    d. Often
    e. Very Often

13. How often do you pray?
    a. Never
    b. Rarely
    c. Occasionally
    d. Often

e. Very Often

14. How often do you experience situations in which you have the feeling that God or something divine intervenes in your life?
    a. Never
    b. Rarely
    c. Occasionally
    d. Often
    e. Very Often

15. Do you have particular dietary preferences?
    a. I am a vegan
    b. I am a vegetarian
    c. I am a semi-vegetarian
    d. I do not have any dietary preference
    e. Other

16. Rate how strongly you agree with the following statements:
    a. *Morally, animals always count for less than humans*

Strongly Disagree	Disagree	Somewhat Disagree	Neither Agree nor Disagree	Somewhat Agree	Agree	Strongly Agree
○	○	○	○	○	○	○

b. *Humans have the right to use animals however they want to*

Strongly Disagree	Disagree	Somewhat Disagree	Neither Agree nor Disagree	Somewhat Agree	Agree	Strongly Agree
○	○	○	○	○	○	○

c. *It is morally acceptable to keep animals in circuses for human entertainment*

Strongly Disagree	Disagree	Somewhat Disagree	Neither Agree nor Disagree	Somewhat Agree	Agree	Strongly Agree
○	○	○	○	○	○	○

d. *It is morally acceptable to trade animals like possessions*

Strongly Disagree	Disagree	Somewhat Disagree	Neither Agree nor Disagree	Somewhat Agree	Agree	Strongly Agree
○	○	○	○	○	○	○

e. *It is morally acceptable for cattle and pigs to be raised for human consumptions*

Strongly Disagree	Disagree	Somewhat Disagree	Neither Agree nor Disagree	Somewhat Agree	Agree	Strongly Agree
○	○	○	○	○	○	○

f. *Chimpanzees should have basic legal rights such as a right to life or a prohibition of torture*

Strongly Disagree	Disagree	Somewhat Disagree	Neither Agree nor Disagree	Somewhat Agree	Agree	Strongly Agree
○	○	○	○	○	○	○

g. It is morally acceptable to perform medical experiments on animals that we should not perform on any human

Strongly Disagree	Disagree	Somewhat Disagree	Neither Agree nor Disagree	Somewhat Agree	Agree	Strongly Agree
○	○	○	○	○	○	○

h. *Pigs should be taken care of by humans just like dogs are*

Strongly Disagree	Disagree	Somewhat Disagree	Neither Agree nor Disagree	Somewhat Agree	Agree	Strongly Agree
○	○	○	○	○	○	○

Appendix B. Demographic data of respondents [most frequent highlighted].

Characteristic	Breakdown	Percentage of total participants
Age	18-24 years	5.1%
	25-34 years	40.4%
	35-44 years	25.8%
	45-54 years	16.9%
	55-64 years	9.0%
	65+ years	2.8%
Gender	Male	41%
	Female	59%
Education level	No formal education, Incomplete secondary schooling	2.3%
	Secondary School graduate	26.4%
	Non-tertiary qualification (diploma, associate degree, etc.), vocational qualification	29.7%
	Undergraduate tertiary qualification	30.3%
	Post-graduate tertiary education	11.2%
Employment status	Employed	75.3%
	Out of work or unable to work	13.0%
	Homemaker	7.3%
	Student	4.5%
Children	Yes	44.9%
	No	55.1%
Ethnic Background	North American	60.1%
	European	28.7%
	Asian, African, South American, Oceanian	11.3%

Appendix C. Respondent Religion, Religiosity, Dietary preference and Speciesism [most frequent highlighted]

Characteristic	Breakdown		Percentage
**Religion**	Yes		53.4%
		Christianity	85.2%
		Buddhism	4.1%
		Hinduism	1.1%
		Judaism	9.6%
	No, I'm an atheist		38.2%
	Not sure		6.2%
	I'd rather not say		2.2%
**Religiosity**	Not Religious (CRS: 1.0 – 2.0)		51.1%
	Religious (CRS: 2.1 – 3.9)		34.8%
	Highly Religious (CRS: 4.0 – 5.0)		14.0%
**Dietary Preference**	Vegan		1.1%
	Vegetarian or semi-vegetarian		14.0%
	No dietary preference		80.3%
	Other		4.5%
**Speciesism score**	Low speciesism		33.1%
	Medium speciesism		65.2%
	High speciesism		1.7%

Appendix D. Significance of differences in agreement between participants given a religious reason or ethical reason [*Significant differences highlighted].

**No treatment or substitute treatment of reduced efficacy or increased expense**	**Agreement score for religious reason (M) ± SD**	**Agreement score for non-religious reason (M) ± SD**
No Treatment	3.91 ± 2.0	3.70 ± 2.0
Substitute Treatment has 10% higher risk of pain	4.35 ± 1.9	3.93 ± 1.9
Treatment that causes 10% higher risk of disability	3.96 ± 2.0	3.41 ± 1.9
Substitute Treatment has small increased risk of death *	4.63 ± 1.9	3.95 ± 2.0
Treatment that causes medium increased risk of death *	4.28 ± 2.0	3.68 ± 2.0
Treatment that causes large increased risk of death *	3.90 ± 2.2	3.17 ± 1.9
$100	4.90 ± 1.9	4.63 ± 1.9
$500	4.39 ± 2.0	4.20 ± 1.9
$1000	4.12 ± 1.9	3.80 ± 1.9
$10,000	3.46 ± 1.9	2.94 ± 1.8
$100,000	2.95 ± 1.9	2.69 ± 1.8
$1,000,000	2.79 ± 1.9	2.28 ± 1.7

Appendix E. Level of agreement to provide substitute treatments of reduced efficacy requested for varying reasons

Question<br>(Reason for requesting a substitute of reduced efficacy)	Agreement score (M) ± SD	Strongly agree or Agree (%)	Strongly Disagree or Disagree (%)
Jewish parents	3.91 ± 2.0	28.7%	34.8%
Christian parents	3.85 ± 2.1	31.5%	37.7%
Muslim parents	4.04 ± 2.0	31.4%	32.0%
Vegan parents	3.39 ± 2.0	22.4%	47.1%
Parents with an irrational belief	2.90 ± 1.9	16.9%	59.6%

Appendix F. Level of agreement to provide substitute treatments of increased expense requested for varying reasons

Question<br>(Reason for requesting a substitute of increased expense)	Agreement score (M) ± SD	Strongly agree or Agree (%)	Strongly Disagree or Disagree (%)
Jewish parents	4.88 ± 1.7	46.0%	12.4%
Christian parents	4.75 ± 1.8	44.4%	16.8%
Muslim parents	4.84 ± 1.8	44.9%	14.6%
Vegan parents	4.41 ± 1.9	37.0%	22.5%
Parents with an irrational belief	3.86 ± 2.0	28.7%	35.4%

Appendix G. Level of agreement to prolong futile treatment for a religious or non-religious reason

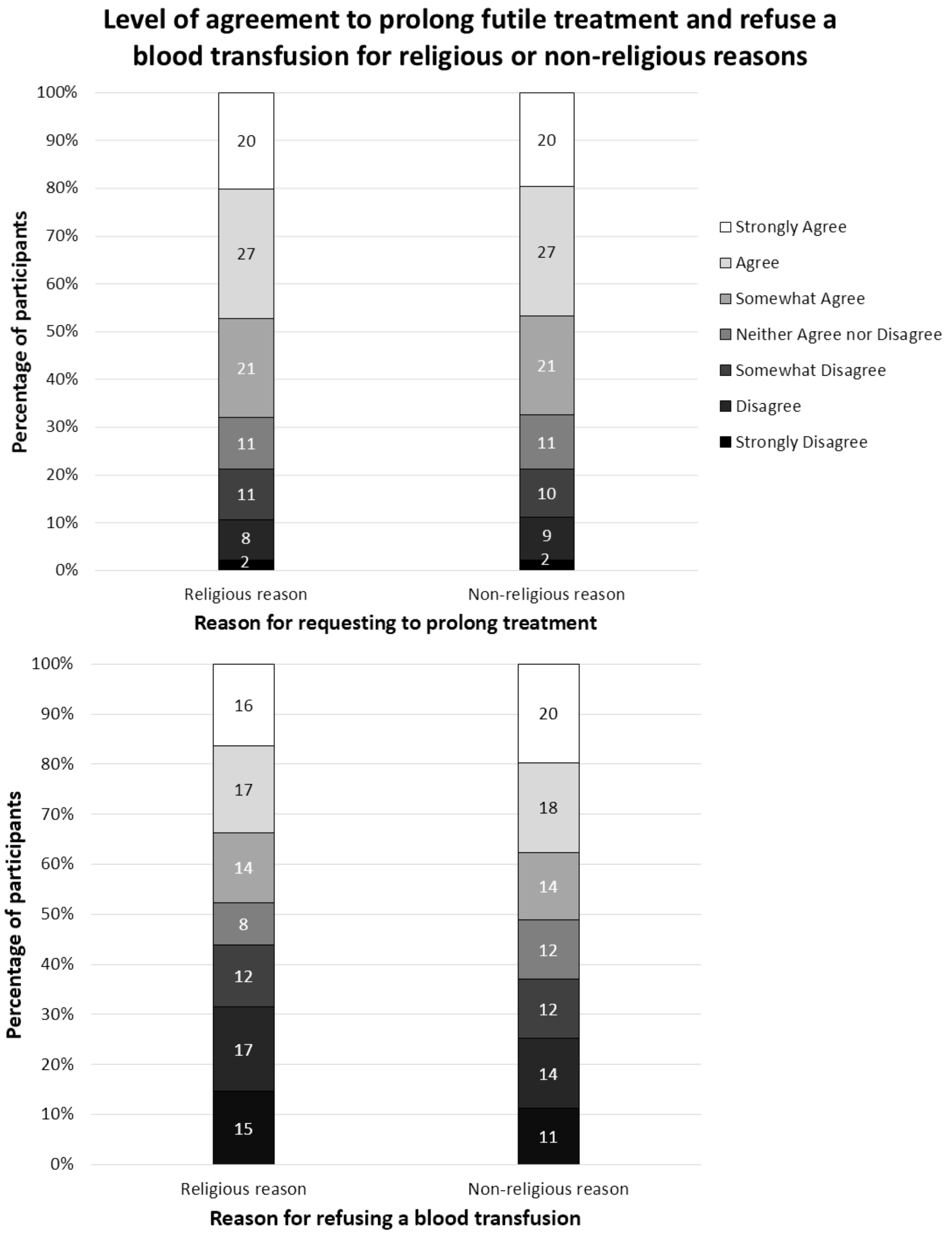

Appendix H. Comparison of agreement scores between Highly Religious and Not Religious participants

Scenario	Reason	Not Religious respondents' agreement scores (M) ± SD N=91	Highly Religious respondents' agreement scores (M) ± SD N=25
Choosing a substitute with reduced efficacy	Jewish parents	3.70 ± 1.9	4.40 ± 2.0
	Christian parents	3.67 ± 2.1	4.60 ± 2.0
	Muslim parents	3.78 ± 2.0	4.56 ± 1.9
	Vegan parents	3.13 ± 2.0	3.88 ± 2.2
	Parents with an irrational belief	2.70 ± 1.9	3.76 ± 2.1
Choosing a more expensive substitute	Jewish parents	4.79 ± 1.7	5.12 ± 1.7
	Christian parents	4.53 ± 1.9	5.32 ± 1.6
	Muslim parents	4.77 ± 1.8	5.00 ± 1.8
	Vegan parents	4.33 ± 2.0	4.52 ± 2.2
	Parents with an irrational belief	3.74 ± 2.0	4.44 ± 2.0
Prolonging futile treatment	Religious	4.78 ± 1.7	5.24 ± 1.8
	Non-religious	4.89 ± 1.7	5.16 ± 1.9
Refusing a blood transfusion	Religious	4.38 ± 2.0	3.64 ± 2.3
	Non-religious	4.55 ± 1.9	3.80 ± 2.3
